# Does Cardiorespiratory Fitness Modify the Association between Birth Weight and Insulin Resistance in Adult Life?

**DOI:** 10.1371/journal.pone.0073967

**Published:** 2013-09-17

**Authors:** Tomoko Aoyama, Kazuyo Tsushita, Nobuyuki Miyatake, Takeyuki Numata, Motohiko Miyachi, Izumi Tabata, Zhen-Bo Cao, Shizuo Sakamoto, Mitsuru Higuchi

**Affiliations:** 1 Graduate School of Sport Sciences, Waseda University, Tokorozawa, Saitama, Japan; 2 Japan Society for the Promotion of Science, Chiyoda, Tokyo, Japan; 3 Aichi Comprehensive Health Science Center, Chita, Aichi, Japan; 4 Department of Hygiene, Faculty of Medicine, Kagawa University, Kita, Kagawa, Japan; 5 Okayama Southern Institute of Health, Okayama Health Foundation, Kita, Okayama, Japan; 6 National Institute of Health and Nutrition, Shinjuku, Tokyo, Japan; 7 Faculty of Sport and Health Science, Ritsumeikan University, Kusatsu, Shiga, Japan; 8 Faculty of Sport Sciences, Waseda University, Tokorozawa, Saitama, Japan; College of Tropical Agriculture and Human Resources, University of Hawaii, United States of America

## Abstract

**Objective:**

Lower birth weight is associated with higher insulin resistance in later life. The aim of this study was to determine whether cardiorespiratory fitness modifies the association of birth weight with insulin resistance in adults.

**Methods:**

The subjects were 379 Japanese individuals (137 males, 242 females) aged 20–64 years born after 1943. Insulin resistance was assessed using a homeostasis model assessment of insulin resistance (HOMA-IR), which is calculated from fasting blood glucose and insulin levels. Cardiorespiratory fitness (maximal oxygen uptake, VO_2_max) was assessed by a maximal graded exercise test on a cycle ergometer. Birth weight was reported according to the *Maternal and Child Health Handbook* records or the subject’s or his/her mother’s memory.

**Results:**

The multiple linear regression analysis revealed that birth weight was inversely associated with HOMA-IR (β = −0.141, p = 0.003), even after adjustment for gender, age, current body mass index, mean blood pressure, triglycerides, HDL cholesterol, and smoking status. Further adjustments for VO_2_max made little difference in the relationship between birth weight and HOMA-IR (β = −0.148, p = 0.001), although VO_2_max (β = −0.376, p<0.001) was a stronger predictor of HOMA-IR than birth weight.

**Conclusions:**

The results showed that the association of lower birth weight with higher insulin resistance was little modified by cardiorespiratory fitness in adult life. However, cardiorespiratory fitness was found to be a stronger predictor of insulin resistance than was birth weight, suggesting that increasing cardiorespiratory fitness may have a much more important role in preventing insulin resistance than an individual’s low birth weight.

## Introduction

Low birth weight, a proxy for fetal underdevelopment, is associated with increased risk of insulin resistance [1], [2], [3], [4], [5], type 2 diabetes [6], and metabolic syndrome [1], [5] in adults. Insulin resistance is the major metabolic disorder that is a potentially underlying physiological factor for type 2 diabetes and metabolic syndrome [7]. Type 2 diabetes as well as metabolic syndrome has become a major health problem worldwide, and the identification of lifestyle factors able to improve the insulin resistance accompanied by low birth weight is important to achieve primary prevention of these metabolic disorders.

Although a sedentary lifestyle and obesity, especially abdominal obesity, are known to be important in the development of insulin resistance [7], recently cardiorespiratory fitness (CRF) has been recognized as an important predictor of insulin resistance [8], [9]. However, only one study by Laaksonen et al. (2003) has investigated whether CRF in adult life can improve insulin resistance associated with low birth weight [5], and the investigators demonstrated that higher levels of CRF modify the association between low size at birth, as assessed by the ponderal index (kg/m^3^), with insulin resistance, which was estimated using a validated insulin sensitivity index (QUICKI) in middle-aged men. It remains unclear whether CRF modifies the association of birth weight with insulin resistance in other racial groups. Because mean birth weight is lower in the Asian population [10] compared with Westerners [11], [12], [13], the relative contributions of birth weight and CRF to insulin resistance in Asians may differ from those found in the previous study [5]. The aim of this study was to examine the association of birth weight with insulin resistance and to assess how the association is influenced by CRF in Japanese adults, whose birth weights are generally much lower than those of Westerners [14].

## Methods

### Subjects

The subjects were 379 Japanese individuals aged 20–64 years who had taken part in our previous research (the Physical Activity and Fitness for Health Promotion Study) [15], [16]. This cross-sectional study of CRF and health status was conducted between 2007 and 2009 in the following independent institutions in Japan: Waseda University (WU), Aichi Comprehensive Health Science Center (ACHSC), Okayama Southern Institute of Health (OSIH), and the National Institute of Health and Nutrition (NIHN). None of the participants had been diagnosed with diabetes mellitus or were taking any medications that could affect the study variables (i.e., antihypertensive, antilipemic, or diabetes drugs or drugs that alter the energy metabolism).

### Ethics Statement

The research project was approved by the Ethical Committee of WU, ACHSC, OSIH, and NIHN, respectively. Written informed consent was obtained from each participant.

### Birth Weight

The subjects’ birth weights were obtained using a questionnaire-by-mail-survey between 2010 and 2011. We sent questionnaires to 968 participants born after 1943 out of 1,020 participants who enrolled in the above-described research. Data on their CRF, obesity measures, insulin resistance, and confounding variables were recorded in the above-described research, which had been stored at each institution. The questionnaire along with a detailed explanation of the project as described below was sent to 968 participants, using reply-paid envelopes. The subjects were asked to provide their birth weights through the *Maternal and Child Health Handbook* (including the *Handbook of Mothers and Children* and the *Pregnant Women and Nursing Mothers Notebook*) records. The *Maternal and Child Health Handbook* is a unique tool that is provided to women in Japan who report their pregnancies to each municipal office. In Japan, birth weights have been recorded since July 1942, when the *Pregnant Women and Nursing Mothers Notebook* was distributed. Starting in 1948, the *Handbook of Mothers and Children* was distributed instead of the *Notebook*. In 1967, the *Maternal and Child Health Handbook* had taken the place of the *Handbook of Mothers and Children*. Those subjects who had lost the *Handbook* or *Notebook* were asked to report their birth weights from memory if the subject or his/her mother knew the subject’s birth weight precisely. The subjects’ reply rate was 41% (397/968). After excluding questionnaires that were not answered completely (n = 18), the completed questionnaires of 379 subjects (137 males and 242 females) were selected for analysis. Of these subjects, 213 (71 males and 142 females) had *Notebook* or *Handbook* records of their birth weight, and 166 (66 males and 100 females) were able to recall their birth weight or obtain it from their mother.

### Obesity Measures

Body mass index (BMI) was calculated from measured height and weight (kg/m^2^). Abdominal circumference was measured at the umbilical region with an inelastic measuring tape at the end of normal expiration.

### Cardiorespiratory Fitness

CRF was assessed by a maximal graded exercise test on a cycle ergometer [Lode Excalibur (OSIH) and Lode Corival (ACHSC), Lode BV, Groningen, Netherlands; Monark Ergomedic 828E (WU, NIHN), Varberg, Sweden] and quantified in terms of maximal oxygen uptake (VO_2_max). The initial work load was 30–60 W, and the work rate was increased thereafter by 15 W/min until the subject could not maintain the required pedaling frequency (60 rpm) [15]. Heart rate and a rating of perceived exertion (RPE) were monitored throughout the exercise. During the progressive exercise test, the expired gas of the subjects was collected, and the rates of oxygen consumption (VO_2_) and carbon dioxide production (VCO_2_) were measured over 30-s intervals using an automated breath-by-breath gas analyzing system [Aeromonitor AE-280S (ACHSC, WU), Minato Medical Science, Tokyo, Japan; Oxycon Alpha (OSIH), Mijnhardt b.v., The Netherlands]. The expired air was collected in Douglas bags, and an O_2_ and CO_2_ mass spectrometer (Arco-1000, Arco System, Japan) was used to analyze the O_2_ and CO_2_ concentrations (NIHN). The volume of expired air was determined using a dry gas volume meter (DC-5, Shinagawa Seisakusyo, Japan) and converted to standard temperature, pressure and dry gas. During the latter stages of the test, each subject was verbally encouraged by the test operators to give maximal effort. VO_2_max was considered to have been achieved if at least two of the following four criteria were achieved: the VO_2_ curve showed a leveling off, the subject’s maximal heart rate was >95% the age-predicted maximal heart rate (220 − age), the respiratory exchange ratio exceeded 1.0, and the subject achieved an RPE of 19 or 20.

### Insulin Resistance

Insulin resistance was assessed using the homeostasis model assessment of insulin resistance (HOMA-IR), calculated as fasting blood glucose (mg/dL) × fasting insulin (µU/mL)/405. Fasting venous blood samples were collected after a fast of 12 h or more, and the concentrations of blood glucose and insulin were measured [16].

### Statistical Analysis

Several confounding variables were included in the analyses. In the general population insulin resistance is associated with hypertension, higher concentration of plasma triglycerides, and lower concentrations of high density lipoprotein (HDL) cholesterol [17]. Therefore, we included the following confounders in the analyses: mean blood pressure [diastolic blood pressure+(systolic blood pressure − diastolic blood pressure)/3], triglycerides, HDL cholesterol [16]. Number of cigarettes smoked per day assessed by means of a questionnaire [16] was also included among the confounders, because smoking may directly increase insulin resistance [18].

Measured and calculated values are presented as means ± SD. HOMA-IR was log-transformed due to non-normal distribution in the analyses. Student’s t-test was used to determine the differences between males and females. Partial correlation analysis was used to test the associations between study variables controlled for gender and age as covariates. Multiple linear regression analyses were performed to assess the associations of birth weight and CRF with insulin resistance. We first entered the birth weight as an independent variable and HOMA-IR as a dependent variable. We then added VO_2_max as an independent variable to examine how the association is altered by CRF. All models were adjusted for gender, age, one of two obesity measures (i.e., BMI or abdominal circumference), study location, mean blood pressure, triglycerides, HDL cholesterol, and smoking status. We also included an interaction term of CRF × birth weight. Multicollinearity in the regression model was assessed by examining the variance inflation factor. All statistical analyses were completed using SPSS 20.0J for Windows (IBM Japan Inc., Tokyo, Japan). The statistical significance level was set at p<0.05.

## Results

The characteristics of the subjects are shown in Table 1. The average age of the subjects was 40.0±10.5 years in males and 41.7±10.7 years in females. The average birth weight was 3.17±0.42 kg in males and 3.11±0.45 kg in females. Significant differences between males and females were observed in most variables, so all models were adjusted for gender, as well as age in the subsequent analyses. Table 2 shows the partial correlation matrix of birth weight and current height, weight, obesity measures (BMI and abdominal circumference), VO_2_max, and HOMA-IR controlled for gender and age as covariates. Birth weight was correlated positively with current height (r = 0.28, p<0.001) and weight (r = 0.13, p = 0.010) and inversely with HOMA-IR (r = −0.15, p = 0.004). Both BMI and abdominal circumference were significantly correlated with HOMA-IR (r = 0.38, p<0.001, respectively). VO_2_max was inversely correlated with HOMA-IR (r = −0.38, p<0.001).

**Table 1:** Characteristics of the subjects.

Variables	All (n = 379)		Males (n = 137)	Females (n = 242)	p-value
Age (yrs)	41.1±10.7	(20–64)	40.0±10.5	41.7±10.7	0.148
Birth weight (kg)	3.13±0.44	(1.80–4.25)	3.17±0.42	3.11±0.45	0.185
Height (cm)	163.4±8.0	(143.0–187.0)	171.0±6.0	159.1±5.5	**<0.001**
Weight (kg)	58.7±9.7	(38.0–99.4)	67.1±8.2	53.9±6.8	**<0.001**
BMI (kg/m^2^)	21.9±2.6	(15.2–31.5)	22.9±2.2	21.3±2.5	**<0.001**
Abdominal circumference (cm)	78.0±8.0	(60.0–104.9)	80.9±7.1	76.3±8.1	**<0.001**
VO_2_max (mL/kg/min)	33.3±7.4	(18.0–56.0)	38.5±7.2	30.4±5.8	**<0.001**
Systolic blood pressure (mmHg)	116.5±13.7	(83–158)	123.2±12.4	112.6±12.8	**<0.001**
Diastolic blood pressure (mmHg)	71.3±10.5	(48–107)	76.2±10.5	68.5±9.4	**<0.001**
Mean blood pressure (mmHg)	86.4±10.9	(60–120)	91.9±10.3	83.2±10.0	**<0.001**
Triglyceride (mg/dL)	48.9±24.3	(10–99)	46.6±28.4	50.2±21.5	0.196
HDL cholesterol (mg/dL)	64.0±15.7	(10–98)	59.1±12.8	66.7±16.5	**0.012**
Fasting blood glucose (mg/dL)	88.8±7.5	(59–118)	91.5±7.3	87.2±7.1	**<0.001**
Fasting insulin (µU/mL)	4.6±2.3	(1.0–14.0)	4.9±2.5	4.5±2.2	0.092
HOMA-IR	1.03±0.55	(0.09–3.50)	1.12±0.61	0.97±0.50	**0.009**

Data are means ± SD (Range).

BMI, body mass index; VO_2_max, maximal oxygen uptake.

**Table 2:** Partial correlation matrix controlled for gender and age as covariates.

Variables	Height (cm)	Weight (kg)	BMI (kg/m^2^)	Abdominal circumference (cm)	VO_2_max(mL/kg/min)	HOMA-IR
Birth weight (kg)	**0.28** ***	**0.13** *	−0.03	−0.01	−0.01	−**0.15** **
Height (cm)	–	**0.48** ***	−0.10	**0.17** ***	0.00	−0.04
Weight (kg)		–	**0.82** ***	**0.76** ***	−**0.17** ***	**0.30** ***
BMI (kg/m^2^)			–	**0.76** ***	−**0.19** ***	**0.38** ***
Abdominal circumference (cm)				–	−**0.32** ***	**0.38** ***
VO_2_max (mL/kg/min)					–	−**0.38** ***

HOMA-IR was log-transformed for analysis.

BMI, body mass index; VO_2_max, maximal oxygen uptake.

* p<0.05;

** p<0.01;

*** p<0.001.

Table 3 presents the results of multiple linear regression analyses of HOMA-IR as a dependent variable. As shown in the model 1, birth weight was inversely associated with HOMA-IR even after adjusting for gender, age, BMI, study location, mean blood pressure, triglycerides, HDL cholesterol, and smoking status (β = −0.141, p = 0.003). Further adjustment for VO_2_max made little difference in the relationship between birth weight and HOMA-IR (β = −0.148, p = 0.001) (model 2). Adjusting for abdominal circumference instead of BMI showed similar results (β = −0.152, p = 0.001) (model 3). This association was largely unaffected by further adjustment for VO_2_max (β = −0.159, p<0.001) (model 4). In the final models (model 2 and 4), gender, age, and obesity variables (BMI or abdominal circumference) were found to be significant predictors of HOMA-IR (gender: p = 0.004, age and BMI: p<0.001 in model 2, gender: p = 0.009, age and abdominal circumference: p<0.001 in model 4). No significant associations were observed between an interaction term (CRF × birth weight) and HOMA-IR (p = 0.997 in model 2, p = 0.857 in model 4).

**Table 3:** Multiple linear regression analysis with HOMA-IR as the dependent variable.

	Independent variables	B	SE	B	p-value	R^2^
**Model 1**	Birth weight (kg)	−0.071	0.024	−0.141	0.003	0.23
**Model 2**	Birth weight (kg)	−0.074	0.023	−0.148	0.001	0.31
	VO_2_max (mL/kg/min)	−0.011	0.002	−0.376	<0.001	
**Model 3**	Birth weight (kg)	−0.076	0.024	−0.152	0.001	0.23
**Model 4**	Birth weight (kg)	−0.079	0.023	−0.159	<0.001	0.29
	VO_2_max (mL/kg/min)	−0.010	0.002	−0.330	<0.001	

HOMA-IR was log-transformed for analysis.

Model 1: adjusted for gender, age, study location, mean blood pressure, triglycerides, HDL cholesterol, smoking status, and BMI.

Model 2: As model 1 plus CRF × birth weight.

Model 3: adjusted for gender, age, study location, mean blood pressure, triglycerides, HDL cholesterol, smoking status, and abdominal circumference.

Model 4: As model 3 plus CRF × birth weight.

B, unstandardized regression coefficient; β, standardized regression coefficient; R^2^, coefficient of determination; VO_2_max, maximal oxygen uptake.

When we separately analyzed the subjects with recorded birth weight data (n = 213) and those with recalled birth weight data (n = 166), we found that the β-related birth weight and HOMA-IR were almost the same between two groups in the model including BMI and VO_2_max as covariates (recorded birth weight group: β = −0.149, p = 0.016, recalled birth weight group: β = −0.164, p = 0.021).

## Discussion

This study was performed to examine the association of birth weight with insulin resistance, and how the association is influenced by CRF in adults. Birth weight was found to be significantly and inversely associated with HOMA-IR after adjustment for gender, age, obesity measurement, and other potential confounders (Table 3). This result is in line with previous studies showing that birth weight is a significant predictor of insulin resistance in adults [1], [3], [4], [5]. Insulin resistance is a major metabolic disorder in the early stages of development of type 2 diabetes and metabolic syndrome [7]. As such, low birth weight may be a cause of type 2 diabetes and metabolic syndrome later in life, while genetic and early environmental factors are of importance in the development of insulin resistance. Although the detailed mechanisms by which low birth weight increases insulin resistance are not completely understood, Hales and Barker proposed the “thrifty phenotype hypothesis” [19]. According to this hypothesis, fetal malnutrition and the consequent low birth weight set in motion mechanisms of fetal nutritional thrift and result in the redistribution of blood flow, with selective protection of brain growth [19]. These changes in the mechanisms that maintain glucose tolerance last permanently after birth, and lead to insulin resistance under the condition of a mismatch between intrauterine constraint and a nutritionally rich postnatal environment. The findings of this study provide further support for this hypothesis and further evidence that low birth weight may be linked to insulin resistance in Japanese adults [20].

The present study showed that the association of lower birth weight with insulin resistance was not modified by CRF. This finding is in contrast with the study by Laaksonen et al. [5]. They reported that higher CRF may moderate the association of lower size at birth with insulin resistance in adult life [5]. There are a number of potential reasons for the differences between the present study’s findings and those of the Laaksonen study [5]. We examined the influence of CRF on the associations between birth weight and insulin resistance in men and women aged 20–64 years, whereas the Laaksonen study was performed in middle-aged men aged 42–60 years. However, there were no differences in the association of birth weight and CRF with HOMA-IR between the males and females or between the younger group and the middle-aged group in the present study (date are not shown). Thus the lack of a modifying effect of CRF in the present study is not explained by differences in gender or age. It is possible that the effect of CRF on the association of birth weight with insulin resistance may be overestimated by the lack of adjustment for obesity in the Laaksonen study [5]. We confirmed, however, that CRF did not modify the association of birth weight with HOMA-IR (β = −0.158, p = 0.001) without adjusting for obesity in our data. As such, the presence of the covariate of obesity is not likely to be related to the lack of modifying effect of CRF.

The difference in race may be a potential reason for the discrepancy between our findings and those of Laaksonen et al. [5]. Because type 2 diabetes occurs in Asians, who are less obese than populations in Western countries [21], the association of birth weight with insulin resistance may be stronger in Japanese, whose birth weights are generally much lower than those of Westerners [14]. This might make it difficult to modify the association of birth weight with insulin resistance by CRF in Japanese. The finding in our study is consistent with that of Ridgway et al. (2011), in which the association between low birth weight and HOMA-IR was not modified by aerobic fitness (watts per kilogram FFM) in 9- and 15-year-olds [22]. As in children and adolescents, it is plausible that the lifelong influence of low birth weight on insulin resistance could not be offset by lifestyle modification such as increasing CRF in Japanese adults.

However, it should be noted that CRF had a closer relationship to HOMA-IR than did birth weight. The contribution of birth weight to HOMA-IR (β = −0.148) was relatively small when compared with that of VO_2_max (β = −0.376) in the model 2 (Table 3). CRF in adult life seems to be a more important determinant of the occurrence of insulin resistance than birth weight. Because exercise improves muscular insulin sensitivity through a variety of mechanisms [23], high levels of CRF with regular exercise may have a protective effect against insulin resistance associated with low birth weight. Thus, increasing CRF may have a much more important role in preventing insulin resistance than does an individual’s low birth weight.

Incidentally, macrosomia, defined as a birth weight of 4 kg or more, is known to be attributed in part to a maternal history of diabetes, which increases the risk of type 2 diabetes in adult offspring [24]. In this study, 14 subjects showed macrosomia. Therefore, we also examined the influence of excluding macrosomic participants on the strength of the contribution of birth weight to HOMA-IR (data not shown). The exclusion of 14 participants with birth weights greater than 4 kg reduced the β-related birth weight and HOMA-IR by a small amount, from −0.148 (p = 0.001) to −0.101 (p = 0.031). These data imply that macrosomic participants in this study may strengthen the association of lower birth weight with insulin resistance. The number of babies with a maternal history of diabetes was thought to be small in relation to the number of babies who were heavy due to good fetal nutrition and for other reasons in the general population [25]. Therefore, the association between higher birth weight and higher insulin resistance was regarded as negligible in this Japanese cohort. Thus we did not exclude macrosomia subjects in the analysis.

Recent trends in industrialized countries, such as the USA, Canada [11], Sweden [12], and Norway [13], show that infants are born heavier, with increased mean birth weight and a decline in the prevalence of low birth weight infants (<2.5 kg). By contrast, the mean birth weight of Japanese infants has steadily declined since the 1980s. Mean birth weight has decreased from 3.19 kg in 1980 to 3 kg in 2010 [14]. In line with this, the incidence of low birth weight babies has increased from 5.2% in 1980 to 9.6% in 2010 in Japan [14]. In these circumstances, successful strategies are especially needed to promote physically active lifestyle, which focus on aerobic exercise to increase CRF for children born with a low birth weight. However, this recent trend toward reduced fetal weight in Japan seems to be partially attributable to changes in maternal health, such as an increase in the prevalence of underweight (BMI <18.5 kg/m^2^) in young women [26] and of low dietary intake during pregnancy [27]. The present study showed that CRF in adult life did not modify the association of birth weight with insulin resistance. In other words, it would be difficult to disentangle the negative influence of low birth weight after birth. Therefore, the Japanese population should be aware of the public health problems associated with low birth weight. Successful strategies are also needed to spread the information among pregnant women that malnutrition of mothers during pregnancy can result in their babies having a future risk of metabolic disorders.

The present study has several limitations. First, as it was a retrospective study, larger sample sizes and long term follow-up studies are needed to confirm the effects of birth weight and adult CRF on insulin resistance. Second, data on gestational age were not available. Nonetheless, adjustment for gestational age has not attenuated the association of thinness at birth with insulin resistance in the previous study [28]. Third, we used both recorded birth weight data and recalled birth weight data (according to subject’s or his/her mother’s memory). However, recalled and recorded birth weight measurements are known to be highly correlated (r = 0.88) [29]. In addition, the association of birth weight and HOMA-IR was similar between the recorded birth weight group and the recalled birth weight group. Fourth, HOMA-IR reflects insulin resistance in the liver rather than in fat and muscle [30], and it is a relatively indirect method of measuring insulin resistance compared with the oral glucose tolerance test (OGTT) and the euglycemic insulin clamp test. Further studies are needed to assess whether birth weight and CRF influence insulin resistance in the whole body, as estimated by the OGTT and euglycemic clamp test. While this remains to be investigated, this study has contributed valuable information pertaining to the Japanese population.

In summary, the present study showed that the association of lower birth weight with higher insulin resistance was little modified by CRF in adult life. However, CRF was a stronger predictor of insulin resistance than was birth weight. These results suggest a lifelong adverse effect of low birth weight on glucose metabolism, as well as an importance of lifestyle factors that can improve insulin resistance. Our data demonstrate that higher levels of CRF would not offset the adverse effects of low birth weight on insulin resistance; however, increasing CRF may have a much more important role in preventing insulin resistance than does an individual’s low birth weight.
